# Prolonged Sitting is Associated with Attenuated Heart Rate Variability during Sleep in Blue-Collar Workers

**DOI:** 10.3390/ijerph121114811

**Published:** 2015-11-19

**Authors:** David M Hallman, Tatiana Sato, Jesper Kristiansen, Nidhi Gupta, Jørgen Skotte, Andreas Holtermann

**Affiliations:** 1 Centre for Musculoskeletal Research, Department of Occupational and Public Health Sciences, University of Gävle, SE 801-76 Gävle, Sweden; 2 Physical Therapy Department, Federal University of São Carlos (UFSCar), São Paulo CEP 13565-905, Brazil; E-mail: tatisato@gmail.com; 3 National Research Centre for the Working Environment, Copenhagen DK-2100, Denmark; E-mails: jkr@arbejdsmiljoforskning.dk (J.K.); ngu@arbejdsmiljoforskning.dk (N.G.); jhs@arbejdsmiljoforskning.dk (J.S.), aho@arbejdsmiljoforskning.dk (A.H.)

**Keywords:** cardiovascular, occupational health, parasympathetic, physical activity, physical inactivity, sedentary

## Abstract

Prolonged sitting is associated with increased risk for cardiovascular diseases and mortality. However, research into the physiological determinants underlying this relationship is still in its infancy. The aim of the study was to determine the extent to which occupational and leisure-time sitting are associated with nocturnal heart rate variability (HRV) in blue-collar workers. The study included 138 blue-collar workers (mean age 45.5 (SD 9.4) years). Sitting-time was measured objectively for four days using tri-axial accelerometers (Actigraph GT3X+) worn on the thigh and trunk. During the same period, a heart rate monitor (Actiheart) was used to sample R-R intervals from the electrocardiogram. Time and frequency domain indices of HRV were only derived during nighttime sleep, and used as markers of cardiac autonomic modulation. Regression analyses with multiple adjustments (age, gender, body mass index, smoking, job-seniority, physical work-load, influence at work, and moderate-to-vigorous physical activity) were used to investigate the association between sitting time and nocturnal HRV. We found that occupational sitting-time was negatively associated (*p* < 0.05) with time and frequency domain HRV indices. Sitting-time explained up to 6% of the variance in HRV, independent of the covariates. Leisure-time sitting was not significantly associated with any HRV indices (*p* > 0.05). In conclusion, objectively measured occupational sitting-time was associated with reduced nocturnal HRV in blue-collar workers. This indicates an attenuated cardiac autonomic regulation with increasing sitting-time at work regardless of moderate-to-vigorous physical activity. The implications of this association for cardiovascular disease risk warrant further investigation via long-term prospective studies and intervention studies.

## 1. Introduction

In recent years, abundant research has been devoted to determining the health effects of inactive behaviors, such as prolonged sitting [1,2]. Prolonged sitting (or sedentary time) is associated with an increased risk for cardiovascular diseases and mortality [3,4,5,6], independently of time spent in moderate-to-vigorous physical activity. However, research into the physiological determinants underlying the relationship between sitting time and cardiovascular health is still in its infancy [7].

Inactivity may increase cardiovascular disease risks due to changes in central pathways involved in autonomic nervous system regulation [8,9,10]. Altered autonomic function, for example, enhanced sympathetic and reduced parasympathetic tones, may predispose individuals to poor cardiovascular health due to reduced baroreflex sensitivity, reduced endothelial function, and hypertension [10]. Experimental studies indicate that prolonged sitting results in alterations of cardiovascular [11,12] and metabolic [13] biomarkers, which corroborates the findings from studies demonstrating autonomic alterations during prolonged bed-rest [14].

Heart rate variability (HRV) is a useful, non-invasive biomarker of intrinsic autonomic cardiac activity [15]. Autonomic dysfunction is reflected in reduced resting HRV (assessed in a supine position during controlled conditions or during nighttime sleep), and is an established risk factor for cardiovascular disease mortality [16,17,18,19]. While several studies have shown a positive association between physical activity and resting HRV [20,21,22], investigations into the relationship between sitting time and HRV are currently lacking.

In the working population, occupational and leisure-time sitting may be differentially associated with cardiovascular health [23], which may be explained by different durations and time-patterns of sitting during work and leisure [24,25,26]. Therefore, it is recommended to discriminate between work and leisure periods when assessing the association between sitting and health outcomes [23,27,28]. Further, because self-reported measures of sitting are less reliable and prone to bias [29,30], objective methods are recommended for measuring sitting time. Thus, in the current study, we used accelerometry for assessing sitting time across several working days, while resting HRV was assessed during nighttime sleep. To obtain a population with a sufficient dispersion in exposure to occupational and leisure-time sitting while avoiding socioeconomic confounders, the present study was conducted on blue-collar workers.

The aim of the study was to determine the extent to which occupational and leisure-time sitting are associated with nocturnal HRV in blue-collar workers. We hypothesize that objectively measured sitting time is negatively associated with nocturnal HRV.

## 2. Materials and Methods

### 2.1. Study Population and Design

The present study is a part of the “New method for Objective Measurements of physical Activity in Daily living (NOMAD)” study in Denmark, which is a field study employing a short-term, prospective design with continuous, objective measurements of physical activity and heart rate across several days. The design and methods have previously been described in more detail in [28,31]. The study was approved by the regional Ethics Committee in Copenhagen, Denmark (H-2-2011-047) and conducted in accordance with the Declaration of Helsinki. All subjects provided their written informed consent prior to participation.

Data collection was carried out from October 2011 to April 2012. Workers from occupations with varying biomechanical exposures at work (*i.e.*, construction workers, cleaners, garbage collectors, manufacturing workers, assembly workers, health care workers, and mobile plant operators) were recruited from different workplaces, which were selected by convenience, mainly through contact with trade unions or safety representatives. Workplaces were considered eligible if the workers were allowed to participate in the study during working hours.

Inclusion criteria to participate were reporting blue-collar work (>20 hours per week) as the main occupation, and to be between 18 and 65 years old. Exclusion criteria were predominantly white-collar work, sickness absence, current fever, pregnancy, pacemaker, and/or skin allergy to adhesives.

In total, 358 blue-collar workers were invited to participate, of whom 259 volunteered to participate. After excluding 36 workers according to the exclusion criteria detailed above, 223 workers completed a questionnaire and were set up with the accelerometers and heart rate monitor. Of the 223, data from 10 workers were excluded as they reported having worked less than four hours per day, and data from 22 workers were excluded for not having valid, objective measurements on a minimum of one full working day. Of the remaining 191 workers, 126 (males n = 70; females n = 56) had acceptable recordings of HRV during valid nighttime sleep (see the HRV section below), as well as complete data on the covariates, and were included in the statistical analysis.

### 2.2. Procedure

Prior to data collection, all workers were invited to attend information meetings where the study was explained. The workers attending the information meetings completed a short questionnaire in which they provided general demographic information and announced their interest to participate in the full study, explained in detail elsewhere [28].

Objective data on sitting time and HRV were then collected for 24 h per day for four consecutive days for each worker; research staff visited the worker at the workplace on the first and last day. On the first day, workers completed a computer-based questionnaire, underwent anthropometric measurements, and were equipped with accelerometers and a heart rate monitors for objective assessment of sitting time and HRV, and a written diary. On the last day, the participants returned the equipment, and the data were downloaded to a computer by the research staff.

The workers were instructed to remove the accelerometers it they had any discomfort, including disturbed sleep, and to report any non-wear times in a diary, along with noting their times for getting up in the morning, starting and ending work, and going to bed. The workers were also instructed to perform a reference measurement in upright standing position for 15 s each day, which were used for accurate detection of sitting periods (see assessment of sitting time below).

### 2.3. Assessment of Heart Rate Variability (HRV)

Inter beat “RR” intervals (IBI) from the electrocardiogram (ECG) were continuously assessed using the Actiheart monitor (CamNtech Ltd, Cambridge, UK), using two ECG electrodes suitable for long-term recordings (Ambu, blue sensor VL-00-S/25) adhered at the V1/V2 and V4/V5 positions, respectively. The Actiheart is a small, water resistant device designed for long-term recordings over multiple days. R-peaks were detected with a sampling rate of 128 Hz, and IBI values were acquired with a resolution of 1 ms using an interpolation algorithm [32]. Beats for which the IBI deviated by more than 15% from adjacent normal beats were classified as abnormal and removed.

HRV indices in both the time and frequency domains were derived from normal-to-normal IBIs using non-overlapping five-minute windows, according to the guidelines outlined by Task Force of the European Society of Cardiology and the North American Society of Pacing and Electrophysiology [15]. The time domain HRV indices calculated were: the root mean squared successive differences of IBIs (RMSSD) and the standard deviation of IBIs (SDNN). The frequency domain HRV indices calculated were: very low frequency power (VLF, 0.003–0.04 Hz), low frequency power (LF, 0.04–0.15 Hz), high frequency power (HF, 0.15–0.4 Hz), and the ratio between LF and HF components (LF/HF).

All frequency domain HRV indices were calculated using a robust period detection procedure (RPD), which has shown superior performance compared to standard Fast Fourier Transform methods [33,34]. In brief, the RPD procedure forms a set of normalized sine and cosine functions, which are used as predictor variables for the IBIs in a multilinear, robust regression model. The regression algorithm uses iteratively reweighted least squares with a bisquare weighting function, which assigns less weight to data points causing high residuals, and zero weight for outliers.

In the present study, HRV was only analyzed during periods classified as *nighttime sleep*, which were detected by combining diary and accelerometer data. IBI five-minute intervals had to meet a number of criteria to be included in the HRV calculations. First, only the sleep periods reported in the diary that occurred between 21:00 and 09:00 were included. Second, data from the first 30 and last 15 min were removed to avoid transient periods such as falling asleep or waking up. Finally, all intervals for which trunk movement occurred or the posture was not classified as “lying” (see the accelerometer measurements below) were removed, as were periods where the percentage of erroneous IBIs was higher than 5%. From the remaining data, the three five-minute intervals with the highest average IBIs were detected, and the mean HRV (across intervals) was determined and averaged across days. All frequency domain variables (VLF, LF, HF, and LF/HF) showed non-normal distributions, so the data were transformed using the natural logarithm (ln VLF, ln LF, ln HF, and ln LF/HF).

### 2.4. Objective Assessment of Sitting Time

Sitting time was measured continuously using two tri-axial accelerometers (Actigraph GT3X, ActiGraph LLC, Florida, USA) placed on the thigh and trunk, as previously described [35]. Tri-axial acceleration data were sampled at 30 Hz. The Actigraph is a small, water resistant device suitable for long-term recordings over multiple days.

The accelerometers were initialized for recording and downloading of data using the Actilife Software version 5.5 (ActiGraph LLC, Pensacola, FL, USA), and signals were further processed and analyzed using a custom-made MATLAB based software, Acti4 (The National Research Centre for the Working Environment, Copenhagen, Denmark and BAuA, Berlin, Germany), which estimates sitting times and various types of physical activity (see below) with a high sensitivity and specificity, both in standardized and free-living conditions [33,36]. Periods of sitting were detected from the two accelerometer outputs based on algorithms presented previously [35,36]. Sitting was defined as a thigh inclination above 45° and a trunk inclination below 45° [35].

Non-wear time was identified when, (a) the software detected a period longer than 90 min with zero acceleration counts, (b) the participant reported non-wear time, or (c) artefacts or missing data were detected by visual inspection.

Sitting time was only analyzed during valid working days. A working day was considered valid if it contained objective measurements >4 working hours and >75% of the average (across days) reported working time. In addition, a working day was only accepted if it included >4 hours of leisure, and >75% of the average (across days) reported leisure time [28,31]. Each working day was split up into periods of work (defined as the time spent working) and leisure (defined as the waking time not spent working). Both the work and leisure periods were detected based on self-reports, which are known to be valid and reliable for this purpose [37]. Data from periods of self-reported sleep (*i.e.*, bed-time) and non-wear were excluded. Prior to the statistical analyses, the total estimated sitting time (hours) was averaged across valid days and expressed as hours per day at work and during leisure.

### 2.5. Objective Assessment of Physical Activity

Physical activity was collected for use as a covariate in the statistical models. Moderate-to-vigorous physical activity was estimated from the accelerometer recordings detailed above and defined as total time (hours) per day spent in walking fast-pace, (*i.e.*, >100 steps/minute) running, cycling, and walking stairs, which were detected according to Skotte *et al.* [35], Stemland *et al.* [36], and Ingebrigtsen *et al.* [38].

### 2.6. Measurement of Individual and Occupational Factors

Several individual factors were collected for use as covariates in the statistical models. Age was determined from the workers’ Danish civil registration numbers, while gender was assessed using self-report. Smoking was measured using the question: *“Do you smoke?”* using four response categories, which were dichotomized into; yes (“yes daily”, “yes sometimes”) and no (“used to smoke”, “I have never smoked”). Body mass index (BMI, kg·m^−2^) was calculated from objectively measured height (cm) and body weight (kg).

Similarly, several work factors were collected. Seniority (months) in the current occupation was measured using the question: “*For how long have you had the kind of occupation that you have now*?”. Influence at work was measured using four items (Cronbach’s Alpha: 0.78) from the Copenhagen Psychosocial Questionnaire [39]: “*Do you have a large degree of influence concerning your work*?”; “*Can you influence the amount of work assigned to you*?”; “*Do you have a say in choosing who you work with*?”; “*Do you have any influence on what you do at work*?”. The five-point response scale ranges from 1 (“always”) to 5 (“never”). After reversing the scale and recoding it to 0–4, the two items for each index were summed up, whereby a higher number indicates more influence and social support, respectively. Lifting and carrying time at work was measured using a single-item from the Danish Work Environment Cohort Survey (DWECS): “*How much of your working time do you carry or lift*?” The six-point response scale ranges from 1 (“almost all the time”) to 6 (“never”) [40]. Working night shifts was assessed using the question: “*What time of the day do you usually work in your main occupation?*” with five response categories, which were dichotomized into; yes (“fixed nights”, “varying work hours with night shifts”) and no (“fixed days”, “fixed evenings”, “varying work hours without nightshifts”, “other”).

Self-reported data on medical diagnoses were also collected. The life-time occurrence of diagnosed diabetes, cardiovascular disease, hypertension, and depression was assessed using the question: “*Have you ever been diagnosed with one or more of these diseases by a doctor?*” using a dichotomous scale (yes, no) for each disease. Regular use of prescribed heart and/or lung medicine was reported by the workers on a dichotomous scale (yes, no).

### 2.7. Statistical Analyses

Multiple linear regression analyses were carried out to determine the crude associations between occupational and leisure-time sitting and each HRV index, respectively (*i.e.*, IBI, RMSSD, SDNN, ln VLF, ln LF, ln HF, and ln LF/HF)—hitherto referred to as the *crude model* data. In these crude models, sitting times at work and during leisure were included as independent variables, while the HRV indices were used as dependent variables. *Primary adjusted models* were constructed using hierarchical regression analyses with step-wise entry of the following covariates: age, gender, BMI, and smoking (step 1); lifting/carrying at work, seniority in the current occupation, influence at work, and total moderate-to-vigorous physical activity (step 2); occupational and leisure-time sitting (step 3). In the primary adjusted models, sitting time variables were entered in the final step to determine their unique association with HRV after accounting for previously established determinants for HRV. All covariates were chosen *a priori* based on theoretical assumptions and empirical findings of their influence on sitting behavior and HRV (e.g., [16,20,41,42,43,44,45]). For each HRV index, regression coefficients (B) and *p*-values were calculated from the crude and primary adjusted model. For each step (steps 1–3), and for each HRV index, the change in adjusted r*^2^* was calculated in order to determine the proportion of explained variance for each added block of variables.

Based on the primary adjusted models described above, two *secondary adjusted models* were developed using repeated regression analyses with the following additional covariates: life-time occurrence of diseases, and prescribed heart/lung medication. In additional secondary analyses, the adjusted primary regression models were performed after excluding workers who reported night-shifts as part of their regular work schedule (n = 17).

Several procedures were used to test the assumptions of multiple linear regression analysis. First, linearity was inspected between the dependent and independent variables using scatter plots. Then, the residuals from each regression model were checked for normality using normal probability plots. Homoscedasticity was checked by plotting sitting time against the residuals obtained from each regression model. Multicollinearity among the independent variables was checked using Pearson’s correlation coefficients and collinearity diagnostics. The regression models indicated no marked deviations from linearity, homoscedasticity, or normality of the residuals, and there were no indications of multicollinearity.

*A priori* power calculations (Statistics Calculators, www.danielsoper.com) indicated that a sample size of 126 subjects would be sufficient in the primary adjusted models for detecting significant associations with an effect size (r^2^) of at least 0.7 with an adequate statistical power (1–β = 0.80). All statistical analyses were carried out using SPSS software, version 20 (IBM). The level of significance (α) was set at *p* < 0.05.

## 3. Results

### 3.1. Descriptive Data

Objectively measured sitting time and nocturnal HRV data were analyzed from 126 blue-collar workers representing eight different occupational groups as follows: health care (total n = 11), assembly (n = 20), cleaners (n = 20), mining and construction (n = 23), manufacturing (n = 30), garbage collecting (n = 16), mobile plant operating (n = 5), and another occupation (n = 1). The occupations differed substantially in gender distributions; female predominance was found in health care (100%), assembly (95%) and cleaning (85%), while male predominance was found in construction (100%), manufacturing (70%), mobile plant operating (100%), and garbage collecting (100%).

Individual and occupational factors, as well as sitting time, physical activity, and medical diagnoses are summarized in Table 1. The mean age of the workers was 45.9 years. Out of the 126 workers, 44% were females, 39% reported to be current smokers, 35% reported a history of medical diagnoses, *i.e.*, diabetes (4%), cardiovascular disease (2%), hypertension (20%), or depression (14%), and 13% reported prescribed heart/lung medicine. Night-shift work was reported by 14% of the workers. The mean number of valid days was 1.9 for sitting time and 2.6 for HRV. On average (SD), the workers worked 8.3 (2.4) h/day, had 9.0 (2.7) h/day of leisure-time, and spent 7.1 (1.3) h/day in bed. The average duration of objectively measured sitting time was 3.1 (range, 0.7–6.6) h/day and 5.9 (range, 2.0–12.3) h/day for occupational and leisure-time, respectively. The workers spent on average 2.0 h/day in moderate-to-vigorous physical activity, with 1.3 (0.7) h/day at work and 0.7 (0.5) h/day during leisure-time. Mean and SD across subjects for HRV indices are presented in Table 2.

**Table 1:** Descriptive information of the blue-collar workers (n = 126) with valid measures of accelerometry and heart rate variability.

Variable	n	Mean	SD
Age (years)		45.9	9.4
Females, n	56		
Smokers, n	49		
Body mass index (kg·m^−2^)		26.4	4.8
Life-time occurrence of medical diagnoses, n			
Diabetes	5		
Cardiovascular disease	3		
Hypertension	25		
Depression/other mental disorder	18		
One or several diagnoses	44		
Prescribed heart/lung medicine, n	16		
Working night-shift, n	17		
Lifting and carrying at work (0 never to 6 almost all of the time)		3.8	1.3
Seniority in the current occupation (months)		173.7	137.2
Influence at work (scale 0–100)		44.6	21.9
Moderate-to-vigorous physical activity (h/day)		2.0	0.8
Number of valid days for accelerometry		1.9	0.8
Number of valid nights for HRV		2.6	1.0
Occupational sitting (h/day)		3.1	1.5
Leisure time sitting (h/day)		5.9	1.9

**Table 2:** Mean and standard deviations (SD) of heart rate variability (HRV) indices during nighttime sleep in blue-collar workers (n = 126).

HRV Index	Mean	SD
IBI (ms)	1069	157
RMSSD (ms)	50	28
SDNN (ms)	56	24
VLF (ms^2^/Hz)	970	877
ln VLF	6.5	0.9
LF (ms^2^/Hz)	915	953
ln LF	6.4	1.0
HF (ms^2^/Hz)	1022	1446
ln HF	6.3	1.2
LF/HF	1.9	2.3
ln LF/HF	0.2	0.9

Abbreviations: IBI, inter beat intervals; SDNN, the standard deviation of IBIs; RMSSD, the root mean squared differences of successive IBIs; LF, low frequency spectral power; HF, high frequency spectral power; LF/HF, ratio between LF and HF components.

### 3.2. Association between Sitting Time and Nocturnal HRV

The primary adjusted regression models showed occupational sitting time had significant negative associations with both time- and frequency domain HRV indices, namely, IBI, RMSSD, SDNN, ln VLF, and ln LF (all *p* < 0.05)—Table 3. The crude regression models (Table 3) showed significant negative associations between occupational sitting time and nocturnal HRV; although significance at the *p* < 0.05 level was reached only for SDNN and ln LF, while IBI and ln VLF were borderline significant (*p* < 0.10). Leisure-time sitting was not significantly associated with any of the HRV indices.

**Table 3:** Crude and primary adjusted * regression models investigating the association between occupational and leisure-time sitting and nocturnal heart rate variability (HRV) in blue-collar workers.

HRV Index	Sitting Variable	Crude Model (*N* = 126)				Primary Adjusted Model (*N* = 126)			
		B	95%CI Low	High	*p*	B	95%CI Low	High	*p*
IBI (ms)	Occupational sitting	−17.00	−35.80	1.81	0.08	−24.24	−46.98	−1.50	**0.04**
	Leisure-time sitting	6.80	−7.97	21.57	0.36	0.41	−14.43	−15.25	0.96
RMSSD (ms)	Occupational sitting	−3.26	0.06	−6.66	0.14	−4.96	−8.88	−1.05	**0.01**
	Leisure-time sitting	0.16	0.91	−2.51	2.83	1.83	−0.73	4.38	0.16
SDNN (ms)	Occupational sitting	−3.03	−5.87	−0.20	**0.04**	−5.07	−8.48	−1.67	**0.00**
	Leisure-time sitting	0.46	−1.77	2.69	0.68	1.10	−1.12	3.33	0.33
ln VLF	Occupational sitting	−0.10	−0.20	0.01	0.06	−0.19	−0.32	−0.05	**0.01**
	Leisure-time sitting	0.03	−0.05	0.11	0.50	0.00	−0.09	0.09	0.98
ln LF	Occupational sitting	−0.13	−0.25	−0.01	**0.03**	−0.18	−0.32	−0.04	**0.01**
	Leisure-time sitting	0.05	−0.04	0.14	0.30	0.07	−0.02	0.17	0.11
ln HF	Occupational sitting	−0.12	−0.26	0.03	0.12	−0.12	−0.27	0.04	0.14
	Leisure-time sitting	0.00	−0.12	0.11	0.95	0.08	−0.02	0.19	0.13
ln LF/HF	Occupational sitting	−0.01	−0.12	0.10	0.91	−0.03	−0.17	0.10	0.62
	Leisure-time sitting	0.06	−0.03	0.14	0.19	−0.01	−0.09	0.08	0.92

* Adjusted for age, gender, BMI, smoking, lifting/carrying at work, seniority, influence at work, and total moderate-to-vigorous physical activity. Note: significant (*p* < 0.05) associations are bold faced. Abbreviations: IBI, inter beat intervals; SDNN, the standard deviation of IBIs; RMSSD, the root mean squared differences of successive IBIs; LF, low frequency spectral power; HF, high frequency spectral power; LF/HF, ratio between LF and HF components.

Table 4 shows the change in adjusted r^2^ during the stepwise addition of the covariates for the primary adjusted model. The proportion of variance (adjusted r^2^) in HRV explained uniquely by the two sitting time variables (step 3) ranged from 2 % for IBI to 6 % for SDNN and ln LF. Overall, the total explained variance (adjusted r^2^) in HRV derived from the model containing all the independent variables (steps 1–3) ranged between 10 % for ln VLF to 31 % for ln HF. The largest proportion of explained variance was attributable to the covariates entered in step 1 (*i.e.*, age, gender, BMI, and smoking); conversely, the additional covariates entered in step 2 (lifting/carrying, seniority, influence, and moderate-to-vigorous physical activity) did not account for any additional variance.

**Table 4:** Proportion of explained variance (adjusted *r*^2^ change) in heart rate variability (HRV) indices for steps 1–3 in the hierarchical regression analyses comprising the primary adjusted model.

HRV Index	Step 1 ^a^	Step 2 ^b^	Step 3 ^c^	Total (Steps 1–3)
	*r*^2^	*r*^2^	*r*^2^	*r*^2^
IBI	0.18	−0.01	0.02	0.19
RMSSD	0.21	0.00	0.05	0.26
SDNN	0.14	0.00	0.06	0.20
ln VLF	0.08	−0.02	0.04	0.10
ln LF	0.19	−0.01	0.06	0.23
ln HF	0.29	0.00	0.02	0.31
ln LF/HF	0.19	−0.02	−0.01	0.16

**^a^** Age, gender, BMI, and smoking; **^b^** lifting/carrying at work, seniority, influence at work, and total moderate-to-vigorous physical activity (in addition to the covariates in step 1); **^c^** occupational and leisure-time sitting (in addition to the covariates in steps 1 and 2). Abbreviations: IBI, inter beat intervals; SDNN, the standard deviation of IBIs; RMSSD, the root mean squared differences of successive IBIs; LF, low frequency spectral power; HF, high frequency spectral power; LF/HF, ratio between LF and HF components.

### 3.3. Secondary Model Analyses

To verify the results from the primary adjusted regression models, secondary adjusted models were run which accounted for prescribed heart/lung medication, and life-time occurrence of medical diagnoses (*i.e.*, diabetes, cardiovascular disease, hypertension, and depression or other mental diseases). These adjustments did only marginally change the associations between occupational sitting time and HRV (IBI *p* = 0.15, RMSSD *p* = 0.03, SDNN *p* = 0.03, ln VLF *p* = 0.11, ln LF *p* = 0.08, ln HF *p* = 0.16, ln LF/HF, *p* = 0.71). Leisure-time sitting remained non-significantly associated with all of the HRV indices (all *p* > 0.05).

To further verify the results from the primary analyses, the adjusted regression models were performed after excluding workers reporting night-shift work (either sometimes or regularly, n = 17). This provided similar significant results for occupational sitting time as for the entire sample (IBI *p* = 0.03, RMSSD *p* = 0.03, SDNN *p* = 0.02, pNN50 *p* = 0.03, ln VLF *p* = 0.03, ln LF *p* = 0.06, ln HF *p* = 0.15, ln LF/HF p=0.75), and similar non-significant associations between leisure-time sitting and HRV (all *p* > 0.05).

## 4. Discussion

### 4.1. The Association between Sitting and Heart Rate Variability

The main finding of this study was that objectively measured occupational sitting time was negatively associated with nocturnal HRV in blue-collar workers. This indicates that increased sitting time at work is associated with decreased autonomic cardiac modulation during nighttime sleep.

There is mounting epidemiologic and experimental evidence for an association between prolonged sitting (or sedentary time) and poor cardiovascular health [1,3,5]. Studies have shown that prolonged sitting can result in elevated blood pressure [12,46], altered endothelial function [11], and changes in metabolic markers [13]. The cardiovascular effects resulting from too much sitting may be attributed to unfavorable changes in autonomic regulation [10,14,16], which can be assessed through HRV [15]. Several studies indicate that a lack of moderate-to-vigorous physical activity is associated with decreased resting HRV in adults, particularly for parasympathetic HRV indices [20,21,22], although negative findings have also been reported [45]. Inactivity induced due to prolonged bed-rest has also been shown to reduce HRV [47], which is a recognized risk factor for cardiovascular diseases and mortality [17,19]. However, it cannot be concluded from previous studies whether sitting time *per se* is associated with resting HRV, and whether this potential relationship exists independently of physical activity.

We used objective measurements of sitting time (during work and leisure) to investigate its association with HRV, while adjusting for objectively measured moderate-to-vigorous physical activity among other covariates. Our novel findings suggest that prolonged sitting (at work) is an important factor for basal HRV in blue-collar workers, regardless of their physical activity levels. Specifically, the reduced time domain HRV indices (IBI and RMSSD) indicate attenuated parasympathetic (vagal) cardiac modulation during nighttime sleep in those workers who spent more time sitting at work. The frequency domain analyses of HRV and SDNN suggest a general reduction in the total power of HRV with increasing occupational sitting. Thus, it is possible that both baroreceptor-sympathetic and parasympathetic (vagal) cardiac modulations are attenuated with increasing occupational sitting time, even if the sympathetic contribution to HRV remains controversial [15,48]. A possible physiologic pathway for the association between prolonged sitting and resting HRV is via inactivity-induced changes in central networks controlling autonomic outflow [14,47]. These central autonomic aberrations may result in systemic cardiovascular changes with reduced baroreceptor sensitivity, increased blood pressure, and blunted endothelial function [10], which may, in turn, predispose individuals to developing cardiovascular diseases.

Occupational sitting explained 6 % of the variance in HRV (SDNN and VLF). Considering that we accounted for multiple individual and occupational factors in the statistical models which are known to be important in autonomic regulation, we therefore believe this proportion of variance can uniquely be attributable to occupational sitting. By comparison, we found that individual factors (age, gender, BMI and smoking) explained between 8 % (VLF) to 29 % (HF) of the variance (between subjects) in HRV, which is in line with previously published values [41,42,45]. We found that occupational factors (lifting/carrying, job-seniority, and influence at work) and objectively measured moderate-to-vigorous physical activity did not account for any additional variability in the HRV indices, which corroborate previous findings [45]. Still, the non-significant association between moderate-to-vigorous physical activity and HRV is in contrast to other studies based on self-reported measures of physical activity [20,21]. Although it is possible that other biomechanical exposures, not captured by the accelerometers or reported by the workers, affected the association between occupational sitting and HRV, we believe the current selection of covariates is adequate.

The association between occupational sitting and HRV did only slightly change with adjustment for the life-time occurrence of medical diagnoses (secondary models), including diabetes, cardiovascular disease, hypertension, and depression; although significance was only reached for RMSSD and SDNN. However, it is still possible that the occurrence of other pre-clinical health conditions which we did not consider, such as perceived work-stress and fatigue, may have confounded this association. For example, work-stress is known to be associated with attenuated HRV [44], and individuals with high levels of stress at work may increase their occupational sitting time [49,50]. Although work-stress was not specifically addressed in the present study, we adjusted for influence at work (decision authority), which is a psychosocial factor of importance for inactive behavior [51]. Also, by performing a secondary analysis with exclusion of workers reporting night-shift work, we accounted (at least to some extent) for potential confounding by the occurrence of general fatigue due to irregular work hours [52,53].

The crude (unadjusted) regression models showed the same trends for the association between occupational sitting and HRV as in the adjusted models, although SDNN and ln LF were the only HRV indices reaching significance. These slightly weaker associations may be explained by HRV being strongly correlated to individual factors (*i.e.*, age, gender, smoking, and BMI), which may have attenuated the associations between sitting time and HRV in the crude regression models.

In contrast to occupational sitting time, leisure-time sitting did not show any significant association with HRV, despite sitting time being higher during leisure than at work. A possible explanation is that the temporal distribution of sitting and non-sitting periods may have been different between work and leisure [24,26], which may have resulted in different health effects of sitting exposure between the two settings. Accordingly, the temporal pattern of sitting has previously shown to be associated with cardiovascular health [11,12]. Still, the possible differential effects of occupational and leisure-time sitting on HRV need to be investigated in further studies.

Our results may contribute to the development of evidence-based guidelines for occupational sitting time, and suggest that reducing occupational sitting may be of importance for cardiovascular health, even in blue-collar workers.

### 4.2. Methodological Discussion

A clear strength of the present study is the simultaneous objective measurement of sitting time and HRV across multiple days. Accelerometry is known as a valid and reliable method for determining sitting time in free-living conditions [36]. Conversely, self-reported measures of sedentary behavior and physical activity have been shown to be less precise and prone to bias [29,30], which may result in erroneous exposure-outcome relationships [54].

Several potential limitations should also be acknowledged. First, the study used a relatively small sample size, which may have resulted in marginal statistical power to detect possible associations with HRV indices of smaller effect sizes (*i.e.*, r^2^ below 0.7). Still, reliability studies of HRV [42,55,56] indicate that the current sample size is adequately powered to detect clinically and biologically relevant changes (across subjects) in most HRV indices. Second, even though we conducted repeated exposures measurements across several days, we could not address the causal relationship between sitting time and HRV. Thus, long-term prospective studies and interventions are needed to confirm whether exposure to prolonged sitting causes changes in HRV. Third, to obtain further information about sleep quality, sleep could have been assessed in more detail. Still, combining diaries with accelerometers is sufficient for the purpose of identifying whether people are sleeping or not [57]. By applying rigorous criteria for the sleep period, and by deriving HRV indices only from supine periods with the lowest average heart rate, we are confident that the HRV measures were not affected by nocturnal movements, arousals, or poor sleep quality to any marked extent. Another possible limitation is that the work and leisure-time periods were determined based on diaries, which may have led to reduced precision compared to momentary assessment.

## 5. Conclusions

We found that objectively measured occupational sitting time is negatively associated with nocturnal HRV in blue-collar workers, independent of many individual and occupational factors known to be associated with HRV, including moderate-to-vigorous physical activity. We suggest further investigation of the association between objectively measured sitting time (at work and during leisure) and HRV via long-term, prospective studies and intervention studies in different occupational groups to assess the potential implications on cardiovascular disease risk.
